# A comparison of two commercial treatment‐planning systems for IMRT

**DOI:** 10.1120/jacmp.v6i3.2054

**Published:** 2005-08-17

**Authors:** M. Peter Petric, Brenda G. Clark, James L. Robar

**Affiliations:** ^1^ Department of Medical Physics, BC Cancer Agency and Department of Physics and Astronomy University of British Columbia 600 West 10th Avenue Vancouver British Columbia V5Z 4E6; ^2^ Department of Medical Physics, Nova Scotia Cancer Centre and Department of Radiation Oncology Dalhousie University 5820 University Avenue Halifax Nova Scotia B3H 1V7 Canada

**Keywords:** intensity‐modulated radiation therapy, treatment‐planning systems, comparison, Varian Eclipse Helios, BrainLAB, BrainSCAN

## Abstract

This study compared the clinical functionality of BrainSCAN (BrainLAB) and Helios (Eclipse, Varian) for intensity‐modulated radiation therapy (IMRT) treatment planning with the aim of identifying practical and technical issues. The study considered implementation and commissioning, dose optimization, and plan assessment. Both systems were commissioned for the same 6 MV photon beam equipped with a high‐resolution multileaf collimator (Varian Millennium 120 leaf). The software was applied to three test plans having identical imaging and contour data. Analysis considered 3D axial dose distributions, dose‐volume histograms, and monitor unit calculations. Each system requires somewhat different input data to characterize the beam prior to use, so the same data cannot be used for commissioning. In addition, whereas measured beam data was entered directly into Helios with minimal data processing, the BrainSCAN system required configured beam data to be sent to BrainLAB before clinical use. One key difference with respect to system commissioning was that BrainSCAN required high resolution data, which necessitated the use of detectors with small active volumes. This difference was found to impact on the ability of the systems to accurately calculate dose for highly modulated fields, with BrainSCAN being more successful than Helios. In terms of functionality, the BrainSCAN system uses a dynamically penalized likelihood inverse planning algorithm and calculates four plans at once with various relative weighting of the planning target and organ‐at‐risk volumes. Helios uses a gradient algorithm that allows the user to make changes to some of the input parameters during optimization. An analysis of the dosimetry output shows that, although the systems are different in many respects, they are each capable of producing substantially equivalent dose plans in terms of target coverage and normal tissue sparing.

PACS number: 87.53.Tf

## I. INTRODUCTION

Over the past 10 years, intensity‐modulated radiation therapy (IMRT) has been the subject of considerable research and development effort. Originally restricted to larger academic centers, IMRT is now being implemented in many centers worldwide. In spite of its potential to improve target coverage and normal tissue sparing, implementation and commissioning of IMRT remain labor‐intensive, and the choice of planning system is a crucial component that may, in some circumstances, substantially impact the time and effort required.

Traditional radiation therapy planning is a manual, iterative, forward process where fields are placed, beam modifiers are inserted, and modifications are made after manual inspection of the dose distribution calculated after each iteration. In contrast, IMRT planning is an inverse process where the required dose distribution over the target and surrounding structures is specified, and an optimization algorithm calculates a 2D intensity pattern (fluence map) for each field to achieve that specification. Most inverse planning algorithms use iterative methods in which thousands of beam profiles are generated and evaluated before arriving at a solution that satisfies the input criteria.^(^ ^1^ ^)^ For each arrangement, a single value cost function, usually defined in terms of irradiation of normal tissue and loss of dose homogeneity over the target, is assessed. At each iteration, the algorithm attempts to reduce this cost function to a minimum. A number of approaches have been developed to define and efficiently minimize cost functions for inverse treatment planning.^(^ ^2^ ^–^ ^6^ ^)^

The degree of success achieved by the optimization process is largely dependent on the cost function used by the algorithm (which in turn depends on the structures defined by the user) and the algorithm used for minimization. While several studies have been carried out to evaluate the various optimization algorithms available,^(^ ^7^ ^–^ ^9^ ^)^ two recent studies have compared commercial IMRT planning systems. A study by Fogliata *et al.* ^(^ ^10^ ^)^ compared the inverse planning algorithms used by three commercial systems. While thorough in its analysis of dosimetric outcome, this study excluded all user considerations such as user interfaces, optimization efficiency, and plan design and evaluation tools. A study by Mayo and Urie^(^ ^11^ ^)^ proposes the use of a systematic benchmark method for comparison and presents results of two commercial systems using two different multileaf collimator (MLC)/beam combinations applied to a carefully designed phantom. Our center has two planning systems capable of inverse planning and will shortly be implementing IMRT for treatments of the head and neck region. This study was undertaken to survey the differences between the two systems in terms of user interface and functionality. Both systems were commissioned for the same MLC/beam combination and were applied to three typical patient CT image data sets.

## II. METHOD

This investigation assessed the BrainSCAN v5.2 (BrainLAB AG, Germany) and Helios (Eclipse v7.1.31, Varian Medical Systems Inc., USA) IMRT planning systems. The BrainSCAN treatment‐planning system includes both the inverse IMRT planning software and the forward planning software, whereas Helios represents only the inverse IMRT planning software component of the Eclipse forward treatment‐planning system. The optimization algorithm employed by BrainSCAN is the dynamically penalized likelihood method,^(^ ^3^ ^)^ while Helios uses a gradient method.^(^ ^5^ ^)^ Using a 6 MV beam (CL21EX, Varian Medical Systems Inc., USA) equipped with a high‐resolution multileaf collimator (Millennium 120 leaf, Varian Medical Systems Inc., USA), these systems were compared in terms of commissioning and implementation, system functionality, and quality of final output plans.

### A. Implementation and commissioning

Both planning systems required specific beam commissioning data before use. A summary of the data required is displayed in Table 1. Differences in the measurement resolutions required by each system necessitated the use of different detector types. Specifically, BrainSCAN's requirement for high‐resolution percentage depth dose (PDD), relative dose factor (RDF), and transverse and radial profile measurements was fulfilled by using detectors with small active volumes. These measurements were performed using an NAC009 miniature thimble ion chamber with an ionization volume of 0.007 cm^3^ (2 mm central electrode, 6.3 mm outside diameter, 3 mm length) as well as diode detectors. All other measurements were acquired using an IC10 ionization chamber (Scanditronix Wellhofer AG, Germany) with an ionization volume of 0.13 cm^3^.

**Table 1:** Commissioning beam data requirements

Parameter	BrainSCAN	Helios
nominal LINAC output	low resolution	
percent depth dose	1 mm depth resolution	5 mm depth resolution
transverse profiles	0.5 mm resolution	2.5 mm resolution
diagonal profiles	0.5 mm resolution	2.5 mm resolution
relative dose factors	$10\times 10\;{\text{mm}}^{2}$ minimum field size	$20\times 20\;{\text{mm}}^{2}$ minimum field size
MLC transmission factor	low resolution	
MLC leaf gap	low resolution	

Following beam commissioning, the output of each system was validated using both 2D film dosimetry and point checks using standard ionization chambers. These validation measurements were performed using IMRT treatment plans for a sample prostate case. Both systems were used independently to create five‐field IMRT plans for this sample case. Field‐by‐field distributions as well as composite distributions were verified using absolute film dosimetry and ionization chamber point checks in low dose gradient regions.

Film dosimetry measurements were carried out using Kodak EDR2 film (Eastman Kodak Inc., USA) placed perpendicular to the beam central axis at 5 cm depth in a light‐tight Solid Water cassette (Gammex RMI Inc., USA). This cassette consists of two 2 cm‐thick solid water slabs sealed along three edges with nylon screws and a rubber O‐ring. To minimize the occurrence of air gaps, the film was removed from its envelope and paper liner and inserted into the cassette under safelight. Film calibration was performed using PDD measurements on the same phantom with the film parallel to the beam central axis. These PDD measurements were delivered at a source‐to‐surface distance of 98.5 cm with a 5 cm×5 cm field size in order to minimize the effects of low energy scattered photons on the film response.^(^ ^12^ ^)^ All films were digitized using a VIDAR VXR‐16 Dosimetry Pro film scanner (Vidar Systems Corp., USA), and conversion to absolute dose was performed using previously verified in‐house film dosimetry software.^(^ ^13^ ^)^

Comparisons with respect to commissioning and implementation were based on complexity and time required for beam data acquisition, processing of this data, and compilation of the data file necessary for input into the planning software.

### B. Effects of commissioning data resolution

The differences in the spatial resolution of the commissioning data requested by both systems prompted an investigation into the effect of these differences on system output. This investigation was carried out using a method similar to that used in a study by Arnfield *et al.,* ^(^ ^14^ ^)^ which explored the use of high‐resolution film dosimetry to improve IMRT dose calculations. In this study, also using Varian's Eclipse treatment‐planning system, Arnfield *et al.* showed that commissioning data acquired using standard methods, in this case a standard ionization chamber with a volume of 0.13 cm^3^, can lead to inaccuracies of up to 20% for IMRT fields with high‐resolution characteristics. In order to compare BrainSCAN and Helios in this respect, the calculated dose distributions from both systems for an IMRT field with high spatial frequency characteristics were compared with the measured dose distribution for this field acquired using absolute film dosimetry.

The comparison was made possible by the ability of the Eclipse treatment‐planning system to import dynamic MLC files. As a result of this functionality, a high spatial frequency field was selected from a BrainSCAN IMRT plan to perform the comparison. The dynamic MLC file for this field was imported into Eclipse where a forward calculation yielded the predicted dose distribution on a Solid Water phantom CT set. The dose distribution in a plane perpendicular to the field at a depth of 5 cm was calculated with a resolution of 1.25 mm and exported using DICOM RT. The corresponding distribution (also perpendicular to the field and at a depth of 5 cm) was obtained from BrainSCAN using the *Export Dose Map for Individual Beams* function in BrainSCAN. The resolution of the BrainSCAN distribution was 1 mm. The same absolute film dosimetry method described in the previous section was employed to measure the dose distribution. This film‐measured distribution was quantitatively compared to both the Eclipse‐ and BrainSCAN‐calculated distributions using both 1D dose profiles and 2D gamma factor analysis.^(^ ^15^ ^)^ The pass/fail criteria for the gamma analysis were a dose difference of 3% of the prescription dose and a distance‐to‐agreement of 3 mm.

### C. Optimization parameters and system functionality

Although both Helios and BrainSCAN make use of dose‐volume histograms (DVHs) in the objective functions to be achieved in plan optimization, the different optimization routines employed by the systems require somewhat different formats for the input parameters to guide the formation of each plan. Both systems required user‐defined calculation grid sizes, fluence map smoothing, and hot beamlet restrictions. These common parameters were found to have minimal impact on the optimization results in both systems. The only input parameter found to have a significant impact on the results was BrainSCAN's *Normal Tissue Expansion* (NTE) option. *Normal Tissue Expansion* allows BrainSCAN users to specify constraints on the tissue surrounding the PTV by defining a structure enclosing the volume given by two margins around the PTV. The first margin specified around the PTV, which has a minimum value of two times the selected PTV grid size, allows for a volume of tissue immediately surrounding the PTV where no restrictions are placed to allow a dose fall‐off from the PTV. The second margin gives the distance from the PTV for the extent of the calculation, and there is an option to make this the outer patient contour. The volume defined between these two margins becomes a structure where a restriction may be placed to force the algorithm to reduce the dose surrounding the PTV within a defined distance. The effect of using NTE on optimization results was systematically investigated following the plan assessment portion of this study.

Following the setting of input parameters, Helios and BrainSCAN differ significantly in terms of system functionality. Both systems have inherent tools and options that are advantageous to the user once the optimization process has begun. Throughout the course of the study, functionality of each system was systematically explored and a qualitative comparison performed.

### D. Plan assessment

To assess the output plans from both systems, three patients treated previously with 3D conformal radiation therapy were selected, two with head and neck cancer and one with prostate cancer. The choice of sample patients was made to assess and illustrate the ability of the planning systems to accommodate different target/normal tissue combinations. Case 1 was selected to assess the ability to provide parotid sparing for a nasopharynx treatment. Case 2 was selected having a relatively large planning target volume (PTV) that could not be covered using conventional conformal planning to assess the ability of IMRT to obtain coverage. Case 3 required the sparing of an organ‐at‐risk (OAR) directly in contact with the PTV.

Image sets together with target and OAR contours for the patients were entered into both planning systems using DICOM transfer protocols. To establish benchmark parameters, a con‐formal plan giving the best possible dose distribution using conventional techniques was done by an experienced planner for each patient. Dose‐volume histogram data from these plans were used to establish initial dose constraints for optimization.

The quality of an IMRT plan is determined by the parameters chosen for the optimization, specifically the target dose homogeneity requirements and the OAR constraints. For all cases, the PTV constraints were set to require that 100% of the PTV received a dose of 95%. In BrainSCAN, the second PTV constraint was in the form of a “Desired Dose” which was set to 100%. The BrainSCAN algorithm inherently tries to provide a homogeneous dose distribution over the PTV at this dose. For Helios, the second PTV constraint was a maximum dose set to 105%, giving a dose variation across the PTV of 10%.

While the target dose homogeneity can generally be clearly specified, OAR constraints are not as easily defined, the general principle being that the lower the dose, the better the plan. Most planning systems used for IMRT require the user to gain familiarity with the response of the system to the input parameter variation to obtain optimum results. To ensure that this study was as objective as possible, the planning systems were assessed with identical OAR constraints.

The OAR constraint values for this study were developed in a two‐stage process as follows. Each system was run with an initial set of constraints, and these values were adjusted independently as required to obtain an optimized dose distribution based on both target and organ‐at‐risk DVHs. The resulting set of constraints for the two systems was then compared, a final set derived to give the best case scenario, and these constraints were used, without further modification, to calculate the dose distribution for assessment (Table 2). Output plans from both systems were then assessed in terms of axial doses, DVHs, and number of monitor units (MUs).

**Table 2:** Normal tissue constraints as a percentage of prescribed dose

Case#	Organ‐at‐risk	Volume (%)	Dose (%)
1	brain stem	0, 30, 50	83, 42, 20
	spinal cord	0, 40	83, 15
	left parotid	10, 50, 67	30, 20, 15
	right parotid	10. 50, 67	80, 35, 28
2	brain stem	0, 5, 25, 50	50, 35, 20, 10
	spinal cord	0, 5, 25, 50	70, 60, 20, 5
3	rectum	0, 15, 25, 50	100, 95, 80, 40
	bladder	0, 10, 25, 50	100, 75, 35, 5

### E. Efficiency

Analysis of the required MUs was performed by determining the ratio of the MUs for each IMRT plan to the MUs required by the corresponding 3D conformal benchmark plan. All sliding‐window, dynamic MLC fields from both planning systems consisted of 28 segments. Other parameters known to impact the MU requirements were set as follows: BrainSCAN's *Hot Beamlet Restriction* was set to 150%, and BrainSCAN's *Tongue‐and‐Groove* optimization was set to 20%.

## III. RESULTS

### A. Implementation and commissioning

The beam data required by both BrainSCAN and Helios were similar. Both systems required measurements of the nominal LINAC output, PDDs at various field sizes, transverse and diagonal beam profiles at various depths, RDFs, MLC transmission, and effective leaf gap measurements. As mentioned previously, a key difference between the systems with respect to commissioning is the required measurement resolution. As displayed in Table 1, BrainSCAN requires PDD measurements with a depth resolution of 1 mm (this requirement is relaxed to 5 mm resolution for depths over 50 mm), transverse and radial profile measurements with 0.5 mm resolution, and RDF data for field sizes as small as $10\times 10\;{\text{mm}}^{2}$. In contrast, the resolution required by Helios is 5 mm for PDD measurements and 2.5 mm for transverse and radial profiles while Helios RDF measurements require data for a minimum field size of $20\times 20\;{\text{mm}}^{2}$. The similarity of the data required by both systems caused the time requirements for commissioning to be virtually identical. Although BrainSCAN's smaller minimum field size requirement for RDFs necessitates more measurements, this increase was found to be negligible in terms of commissioning time. In terms of commissioning complexity, the only difference between the systems is BrainSCAN's requirement for a high‐resolution mini‐ionization chamber. In order to increase confidence in the small field measurements acquired with this chamber, a series of “spot check” measurements was performed using a photon diode detector. Diode measurements agreed with mini‐ionization chamber measurements within 0.5%.

Analysis of beam data and compilation of the data file varied between the systems. The BrainSCAN system required that all data be sent to BrainLAB for verification, conversion of radial profiles to radial factors, and compilation of the data into a collimator file that can be directly read by the treatment‐planning system. This process took approximately two days from the time the data was sent to BrainLAB to the receipt of the collimator file. For Helios, beam data was compiled, in‐house, into a file readable by the planning system. This compilation took approximately one day.

Output measurements using film dosimetry and ionization chamber point checks indicated good agreement for the sample prostate IMRT plans from both systems. Maximum dose discrepancies were well below 5% for both field‐by‐field measurements and composite distributions.

### B. Effects of commissioning data resolution

**Figure 1:**
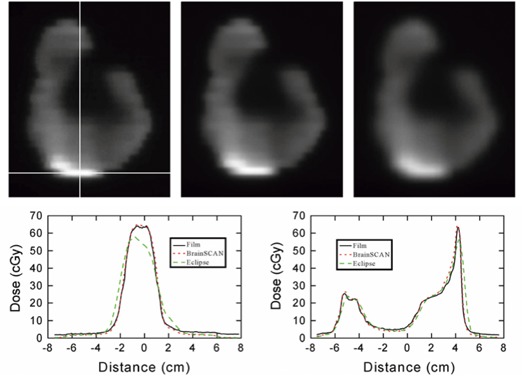
Comparison of measured and calculated fluence distributions from a selected intensity modulated field. The top panel shows the film measurement (left), the BrainSCAN‐ (center), and Eclipse‐calculated distributions (right). The two lower panels show profiles through the three distributions horizontally (left) and vertically (right). The location along which these profiles were obtained is indicated by the white crosshair on the film measurement distribution.

**Figure 2:**
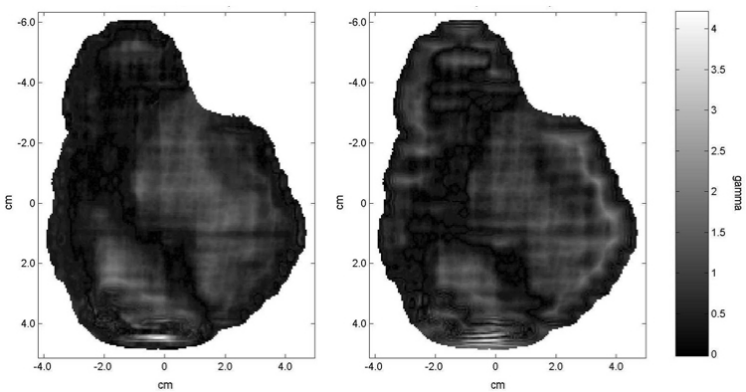
Two‐dimensional gamma maps comparing film‐measured distributions to BrainSCAN (left) and Eclipse‐calculated distributions (right). Analysis was performed within the field edges.

The calculated and film‐measured dose distributions for the selected high spatial frequency IMRT field are displayed in Fig. 1. Also displayed in this figure are 1D dose profiles through the high frequency region of the field. From these profiles it is apparent that there is a difference between the distributions produced by BrainSCAN and Eclipse. The *x* and *y* profiles for the BrainSCAN system show good agreement with the measured data in both low‐ and high‐resolution regions. In contrast, the profiles from the Eclipse distribution show good agreement with measurement in low‐resolution regions, while discrepancies are apparent for high‐resolution areas. At the intersection point of these two profiles, marked by the cross hair in Fig. 1 (x=‐0.32 cm,y=4.19 cm), the percent dose difference from the measured distribution for BrainSCAN was 1.86%, while Eclipse showed a 13.32% dose difference. Results of the 2D gamma factor analysis are displayed in Fig. 2. The low gamma values in these maps indicate that both BrainSCAN and Eclipse show good agreement with the measured data over most of the dose distribution. As expected, the highest gamma values are found in the vicinity of the high‐frequency region of the field. This high‐gamma value region is noticeably larger for the Eclipse‐calculated distribution. The sum of all gamma values, Σγ(i,j), as well as the mean and max gamma values for both gamma factor maps, is displayed in Table 3, further highlighting the dosimetric discrepancies of the Eclipse‐calculated distribution.

**Table 3:** Properties of the masked γ map distributions comparing calculated distributions to film measured distribution

	BrainSCAN	Eclipse
Σγ(i,j)	15863	17029
mean γ (*i,j*)	0.66	0.71
max γ (*i,j*)	3.82	4.22

### C. Optimization parameters and system functionality

Input parameters used to guide the optimization were found to have minimal impact on the optimization results with the exception of BrainSCAN's NTE option. Results indicating the effect of the NTE are shown in the following section.

Once the input parameters are set and the optimization process has begun, BrainSCAN and Eclipse differ significantly in their functionality. The BrainSCAN system calculates four separate plans with different priority on the dose to the OARs. Once all calculation parameters have been chosen by the user, the BrainSCAN system automatically calculates four IMRT plans: *PTV Only, OAR Low, OAR Normal,* and *OAR High.* The *PTV Only* plan is optimized to produce a uniform dose distribution over the volume of the PTV with no consideration for the dose to the OARs. The *OAR Low, OAR Normal,* and *OAR High* plans are optimized with varying priorities placed on the dose to the OARs. The calculation of these plans is accomplished by changing the penalization parameter in the dynamically penalized likelihood algorithm used by BrainSCAN. Once the fluence patterns have been optimized, the dose distributions are calculated and the user is presented with an interface that allows comparison of any two of the plans. The optimal plan is selected based on dose distributions and the DVHs.

Once optimization has begun, the Helios system offers the user the ability to interactively adjust the constraint parameters, and thus the objective functions, while the optimization process is being performed. This allows points on the DVHs to be modified in real‐time during optimization. For example, if the system was observed during optimization to be struggling to achieve a particular important constraint for one of the OARs, the constraints of the other OARs may be adjusted or relaxed until an optimal compromise is achieved. This functionality, combined with the ability to re‐enter and continue optimization at a later time, gives the user direct control over the progression of the optimization.

The two systems also differ in the flexibility offered to define arbitrarily shaped DVHs. Both systems are convenient in that they allow for general DVHs to be saved to a library for repeated use. In BrainSCAN, each DVH is defined using a fixed number of points on a dose‐volume plot. The target DVH is defined by an average dose, set to 100%, and a single point to define coverage of the PTV at a particular dose level. Helios offers the additional option of defining both upper and lower dose limits for the target DVH. The DVHs for the OARs in BrainSCAN are defined by four points on the curves. These points define the upper limits for the resultant DVHs for these organs. In Helios, DVHs can be defined by an arbitrary number of points or by a continuous line drawn by the user. As in BrainSCAN, OAR points define the upper limits for the resultant DVHs for these organs. A unique feature to Helios is the definition of a priority for each point making up the DVH curves for OARs and the PTV. These priorities indicate the relative importance placed on each constraint point and thus provide additional control over the direction of the optimization. In BrainSCAN, the user has the option of adjusting the priority of each OAR relative to the others by specifying *OAR Guardian* values. These values are used to specify the priority of all the DVH points for a given organ but do not distinguish between individual constraint points on the same DVH.

Neither system optimizes gantry or couch angles. Both systems do, however, offer automatic optimization of the collimator angles prior to entering the inverse planning process.

### D. Plan assessment

The optimized dose distributions obtained independently from each system prior to derivation of the final set of optimization constraints were observed to be very similar with no large differences in DVH points between the systems. In all three clinical cases, the final constraints were composed of an equal number of DVH points from the distributions produced by both systems.

**Figure 3:**
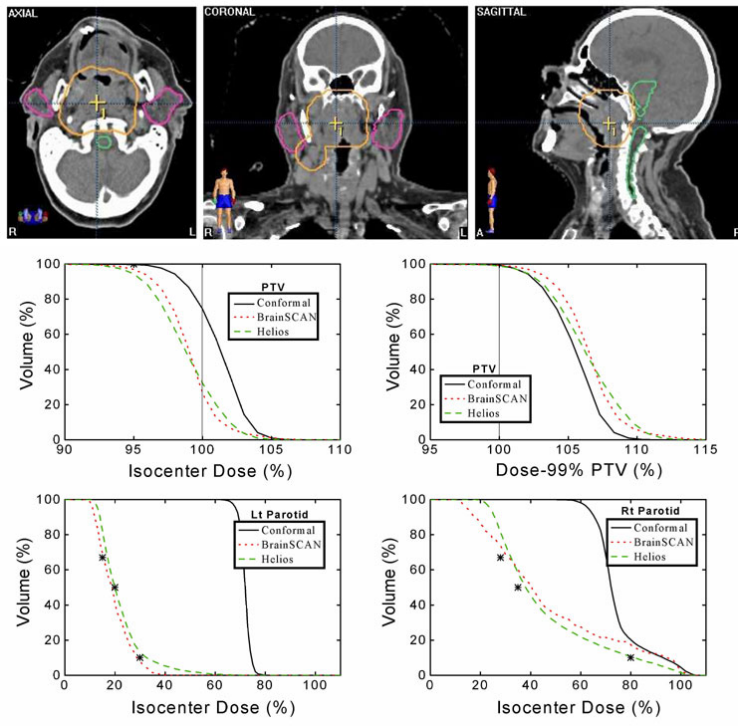
Planning comparison for Case 1. Top: axial, coronal, and sagittal views showing the anatomical relationship between the PTV, right and left parotid glands, and spinal cord. Middle: calculated dose‐volume histograms for the PTV un‐normalized (left) and normalized at 99% coverage of the PTV (right) using a benchmark conformal plan and seven‐field IMRT plans for BrainSCAN and Helios. Bottom: calculated dose‐volume histograms for the parotid glands with all three plans. The asterisks on the dose‐volume histograms indicate the restraint values used for optimization.

Results for Case 1 are shown in Fig. 3. The top panel shows the relatively complex shape of the PTV and the position of the parotid glands. The benchmark conformal plan was composed of one anterior and two lateral wedged fields while both IMRT plans consisted of seven uniformly spaced coplanar beams. The DVH plots illustrate that while neither system produced a plan with PTV coverage as uniform as the conformal plan, both systems were able to achieve substantial sparing of both parotid glands. In addition to displaying the un‐normalized DVH plots for the PTV, this figure also shows the DVHs for the PTV normalized at 99% coverage. Dose‐volume statistics for the PTV in all three cases are displayed in Table 4.

**Table 4:** PTV dose‐volume statistics for all three cases as a function of prescription dose (Rx)

	Case 1		Case 2		Case 3	
	BrainSCAN	Helios	BrainSCAN	Helios	BrainSCAN	Helios
%Vol covered by Rx dose	96.9	94.5	94.6	90.8	96.4	97.0
PTV min (% of Rx dose)	85	87	85	86	76	86
PTV max (% of Rx dose)	107	107	111	106	107	105

**Figure 4:**
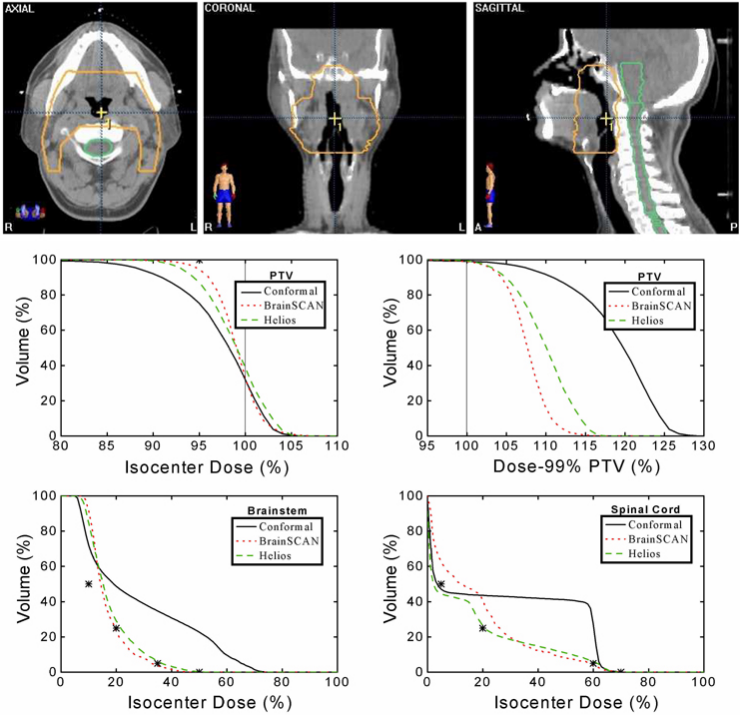
Planning comparison for Case 2. Top: axial, coronal, and sagittal views of Case 2 showing the anatomical relationship between the PTV and spinal cord. Middle: calculated un‐normalized (left) and normalized (right) dose‐volume histograms for the PTV using a benchmark conformal plan and seven‐field IMRT plans for BrainSCAN and Helios. Bottom: calculated dose‐volume histograms for the brainstem and spinal cord with all three plans. The asterisks on the dose‐volume histograms indicate the restraint values used for optimization.

The PTV for Case 2 shown in the top panel of Fig. 4 is a complex horseshoe shape wrapping around both the brainstem and the spinal cord. The benchmark conformal plan was composed of two lateral fixed fields plus two anterio‐lateral 105° conformal arcs while the IMRT plans again consisted of seven uniformly spaced coplanar beams. The DVH plots show that the conformal plan does not provide coverage of the PTV until a dose of approximately 80% of the isocenter dose. Both BrainSCAN and Helios were able to achieve coverage of the PTV by approximately 90% of the isocenter dose with no increase in maximum dose, with BrainSCAN giving a slightly more homogeneous dose (95% to 5% volume change over a 7% variation in dose for BrainSCAN compared to an 11% dose change for Helios). Both IMRT plans also show improvement in dose to the brainstem and spinal cord over the conformal plan with Helios giving a slightly lower dose to the spinal cord in the lower dose range.

**Figure 5:**
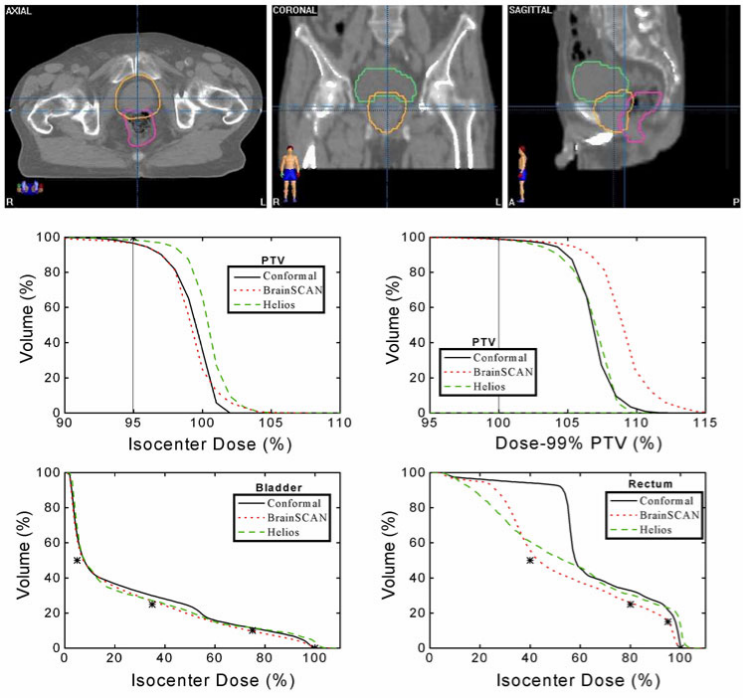
Planning comparison for Case 3. Top: axial, coronal, and sagittal views of Case 3 showing the anatomical relationship between the PTV, bladder, and rectum. Middle: calculated un‐normalized (left) and normalized (right) dose‐volume histograms for the PTV using a benchmark conformal plan and five‐field IMRT plans for BrainSCAN and Helios. Bottom: calculated dose‐volume histograms for the bladder and rectum with all three plans. The asterisks on the dose‐volume histograms indicate the restraint values used for optimization.

Figure 5 shows the results for Case 3, a prostate plan with both bladder and rectum considered as OARs. The benchmark conformal plan was a four‐field box. For this case, the IMRT plans were composed of five uniformly spaced coplanar beams. The PTV DVHs shown in the middle panel illustrate small differences in the three plans. When normalized to the isodose value enclosing 99% of the PTV, it can be seen that the BrainSCAN plan gives increased dose inhomogeneity throughout the PTV with a corresponding small advantage to the rectal dose, mostly at the lower dose values. The bladder dose remains virtually the same in all cases.

**Figure 6:**
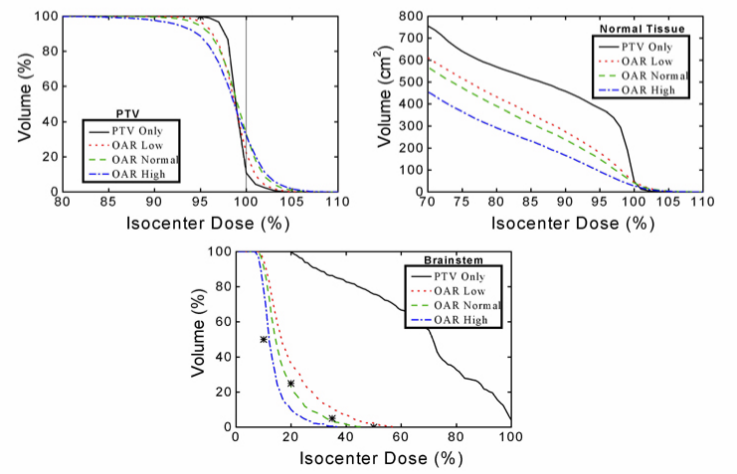
BrainSCAN‐calculated dose‐volume histograms for the PTV, all tissue outside the PTV (Normal Tissue) and the brainstem for Case 2 for the four calculations considering the PTV only and the organs‐at‐risk as a high, normal, and low priority.

A comparison of the results of the four plans displayed by BrainSCAN after each optimization for Case 2 is shown in Fig. 6. The top panel showing the results for the PTV illustrates that while the *PTV Only* option provides the best dose homogeneity, not surprisingly, the addition of the normal tissue and sensitive structure information will degrade this coverage. Clearly, the addition of a constraint considering all tissue outside the PTV, designated “Normal Tissue” and shown in the middle panel, provides for successive tissue sparing outside the PTV. From the bottom panel showing the brainstem results, it can be seen that three of the four dose constraints are met with the *OAR Normal* result.

**Figure 7:**
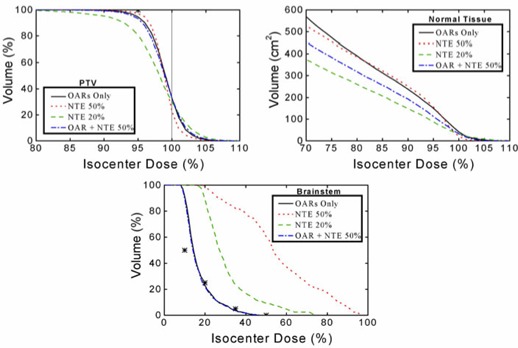
BrainSCAN‐calculated dose‐volume histograms for the PTV, all tissue outside the PTV (Normal Tissue) and the brainstem for Case 2 taking into consideration a volume of risk defined immediately outside the PTV and designated Normal Tissue Expansion (NTE). The four curves in each plot are for the calculations considering the organs‐at‐risk only, the NTE with a maximum dose constraint of 20% and 50% of the isocenter dose and the combination of the organs‐at‐risk and the NTE at the 50% level.

The use of the NTE option adds further flexibility, as illustrated in Fig. 7. For this analysis, an NTE was defined around the PTV extending from 8 mm to 48 mm from the PTV forming an annular volume‐at‐risk with a width of 4 cm. The PTV DVHs shown in the top panel indicate that restricting the maximum dose to this NTE to 20% of the isocenter dose degraded the dose homogeneity and coverage of the PTV. Adding an NTE restriction with a dose maximum of 50% to the calculation including the OARs was almost equivalent to the calculation without the NTE. Considering the normal tissue shown in the middle panel, the addition of the NTE provides tissue sparing. The bottom panel showing the brainstem DVHs shows clearly that the definition of an NTE alone will not provide appropriate structure specific dose sparing.

### E. Efficiency

Results from this analysis are displayed in Table 5. Using BrainSCAN, the increase in required MUs was higher than that for Helios for Case 1 with the two systems showing comparable results for the other cases.

**Table 5:** Ratio of monitor units required to deliver IMRT plans compared to conventional conformal plan

Case	BrainSCAN	Helios
1	2.4	1.7
2	1.7	1.6
3	2.4	2.4
Average	2.2	1.8

## IV. DISCUSSION AND CONCLUSIONS

In terms of implementation and commissioning, dose optimization, and plan assessment, no substantial differences in performance were demonstrated between the BrainSCAN and Helios planning systems. Results indicated that both systems can produce substantially equivalent dose plans in terms of target coverage and normal tissue sparing.

Implementation and commissioning of the systems were found to be identical in terms of complexity and time involved. Acquisition of high‐resolution data for BrainSCAN added only marginally to the overall time required for beam data acquisition. While the requirement to send the commissioning data to BrainLAB added an extra day to the overall commissioning of the BrainSCAN system, it also eliminated the workload of having to compile the beam data file.

While comparable in terms of output plans, BrainSCAN and Helios both have advantages and disadvantages over each other in terms of functionality. Both systems have adequate input mechanisms for dose constraints; however, the BrainSCAN system presents the user with four plans for each optimization from which to choose the optimal. The *PTV Only* option is rarely clinically viable and is done to provide the basis of the subsequent optimization, which includes the OAR constraints. However, the presentation of three calculated plans with slightly different relative constraint weighting provides a quick assessment of the value of making changes in the optimization parameters. We have found this useful in speeding up the software learning process.

Alternatively, Helios offers the option of editing DVH parameters during calculation, thus providing immediate interactive input during the optimization. This feature adds substantial flexibility, provides a good learning tool, and may reduce the overall time for optimization where adequate information for the definition of the constraints is not available *a priori.*

Analysis of the PTV curves shown in Figs. 2 to 4 illustrates one of the difficulties of IMRT planning. With identical constraints set in each case, clearly, there is a frequent requirement for a (small) renormalization of the dose distribution after optimization because the “optimized” plans do not consistently reach the goals set for the PTV. For example, of the three IMRT plans, only the Helios plan in Fig. 5 provides coverage of the PTV at the 95% dose level. There is also a corresponding differing ability of the systems to reach the dose constraints set for the organs‐at‐risk with each system failing to reach a particular constraint in one case or another.

While the analysis of the commissioning requirements of both systems revealed no significant differences in terms of complexity and time requirements, the portion of this study investigating the effects of the differences in commissioning data resolution revealed an inadequacy in the Eclipse treatment‐planning system. Verification measurements on the sample prostate case used during early commissioning revealed no discrepancies larger than 5%. This validation was performed on a typical prostate case with significantly less complex fields compared to the head and neck cases used in the later comparisons. Serious discrepancies as large as 13% were observed when a field with high spatial resolution peaks was analyzed. The large discrepancies observed for this field indicate that the resolution of the commissioning data required by Eclipse is inadequate for dose calculations of high‐resolution IMRT fields. This problem is directly related to the pencil beam kernels used to calculate the dose distributions. These kernels are derived from commissioning data and can be affected by the spatial resolution capabilities of the measuring device used.^(^ ^16^ ^)^

While studies have shown that dose calculations using kernels derived from standard (low resolution) ionization chamber data are accurate for conventional planning scenarios,^(^ ^17^ ^)^ this is not the case for all IMRT fields. IMRT fields can contain highly modulated intensity regions that will be affected by low‐resolution pencil beam kernels.^(^ ^14^ ^)^ The profiles for the Eclipse distribution in Fig. 1 clearly illustrate these effects. These profiles are in good agreement with the film‐measured profiles except in highly modulated regions. In the horizontal profile, Eclipse not only underestimates the dose of the high‐intensity peak by over 13%, but the software also overestimates the horizontal spread of dose at the sides of the peak. The BrainSCAN distribution is in good agreement throughout this profile. In the vertical profile, the narrow peak on the right is again underestimated by the Eclipse system, whereas BrainSCAN is successful in its dose calculation. For the double peak on the left side of this plot BrainSCAN slightly overestimates the dose but is successful in distinguishing the two peaks. Eclipse, on the other hand, is unable to separate the peaks. Although this inadequacy of the Eclipse treatment‐planning system may have a direct impact on the treatment of IMRT plans, it should be noted that this is inherently a problem with the Eclipse forward planning calculation and not with the Helios inverse planning software.

While correction of this problem by directly entering higher resolution data into Eclipse is not possible due to a minimum resolution capability of 2.5 mm, studies have shown that these inaccuracies can be reduced by using high‐resolution film dosimetry to measure the penumbra region of transverse profile commissioning data^(^ ^14^ ^)^ or through direct pencil beam kernel optimization.^(^ ^18^ ^)^ In addition, the clinical impact of this inadequacy is yet to be determined, and it should be reiterated that this effect is only apparent for highly modulated fields. The high‐resolution field used to ascertain the effects of the commissioning data resolution was specifically chosen as being the most highly modulated field produced in the DVH comparison.

In summary, we have demonstrated that both BrainSCAN and Helios have inherent advantages for IMRT planning. Both inverse planning systems are capable of producing substantially equivalent dose plans in terms of target coverage and normal tissue sparing. Several insignificant differences between the systems exist in terms of implementation and commissioning, dose optimization, and plan assessment. One difference brought to light by this comparison was the inadequacy of the Eclipse treatment‐planning system to accurately calculate dose for highly modulated fields. Although this study did not evaluate the clinical impact of this inadequacy, IMRT quality assurance generally has a large impact on time and effort, and discrepancies between calculations and measurements for highly modulated fields will most certainly be problematic.

## ACKNOWLEDGMENTS

The authors express their thanks to BrainLAB AG, Germany, and Varian Medical Systems, U.S.A., for planning system support, and the Natural Sciences and Engineering Research Council of Canada for financial support.
